# Nutritional factors and survival in a cohort of patients with oral cancer

**DOI:** 10.3389/fnut.2025.1530460

**Published:** 2025-03-11

**Authors:** Paolo Buscemi, Cristiana Randazzo, Carola Buscemi, Anna Maria Barile, Elena Finamore, Roberta Caruso, Piero Colombrita, Martina Lombardo, Serena Cangemi, Giulia Zucchi, Adriana Cordova, Antonio Lo Casto, Silvio Buscemi

**Affiliations:** ^1^Sezione di Scienze Radiologiche, Dipartimento di Biomedicina, Neuroscienze e Diagnostica Avanzata (BIND), University of Palermo, Palermo, Italy; ^2^Dipartimento di Promozione della Salute, Materno-Infantile, Medicina Interna e Specialistica di Eccellenza (PROMISE), University of Palermo, Palermo, Italy; ^3^Unit of Clinical Nutrition, Obesity and Metabolic Diseases, University Hospital Policlinico “P. Giaccone”, Palermo, Italy; ^4^Unit of Internal Medicine, “V. Cervello Hospital”, Palermo, Italy; ^5^Chirurgia Plastica e Ricostruttiva, Dipartimento di Discipline Chirurgiche, Oncologiche e Stomatologiche (DICHIRONS), University of Palermo, Palermo, Italy

**Keywords:** mouth neoplasms, cancer of head and neck, malnutrition, body composition, survival

## Abstract

**Background:**

Malnutrition commonly affects patients with oral squamous cell carcinoma (OSCC), which contributes to worsening prognosis. Moreover, specific strategies for diagnosing and managing malnutrition in OSCC are lacking. We aimed to investigate if the modality of nutritional treatment as standard oral (ON) or artificial enteral nutrition (AN), assigned by a dedicated nutritional team or not, influences survival in patients with OSCC. Moreover, given the difficulties in obtaining adequate nutritional evaluation in patients with OSCC we preliminary evaluated the magnetic resonance imaging volumetric reconstruction of posterior paraspinal muscles at the level of the third cervical vertebra (MRI-C3) as a tentative new approach to investigate sarcopenia.

**Methods:**

This retrospective study included 52 consecutive patients with OSCC who underwent surgery at the University Hospital of Palermo (I) from Jan 2020 to May 2023. In-hospital nutritional support was provided by a dedicated team. The patients were retrospectively compared with a control group of 11 patients who were surgically treated for OSCC between January and December 2019 in the same unit of surgery, in the absence of a dedicated nutritional team. Upon discharge, the nutritional treatment consisted of ON or AN. In 2020–2023, industrially produced special medical food formulations were used rather than natural foods as it was in use in 2019; also, adequate protein supplements were assigned in order to reach the recommended protein intake of 1–0-1.2 g/kg ideal body weight. The MRI-C3 volumetric reconstruction was obtained in 16 patients.

**Findings:**

As per-protocol, the patients were categorized according to pre-surgery TNM stage: groups A (TNM stages I-III) and B (IVa-IVc). The prevalence of group A patients was 59.6% in the case group and 85.7% in the control group (*p* < 0.001), with higher survival rates at follow-up in the control group (80.5% vs. 27.3%), therefore, the two historical groups were not comparable. Given the limited number of cases, all patients were included in a unique group. Advanced stages of OSCC (log-rank test, *p* < 0.001) and AN (*p* < 0.001) were independently associated with a lower survival rates. The 3–12 month post-surgery MRI_C3 volume increased in patients who received ON treatment and decreased in those who received AN treatment (*p* < 0.001).

**Conclusion:**

AN is associated with lower survival probability than ON in patients with OSCC. The MRI-C3 measurement of paravertebral muscles is a promising technique for detecting sarcopenia that needs to be confirmed by further studies including larger groups of patients.

## Introduction

Data obtained from Globocan 2020 estimate that head and neck squamous cell carcinoma is the seventh most common cancer (1). Similar to what has been observed in the European Union, in Italy, the annual incidence of head and neck cancers is 18/100,000 inhabitants; about 93% of these cancers are epithelial tumors, and 90% are malignant squamous cell carcinomas (2, 3). Malnutrition is characterized by the depletion of energy, protein reserves, and other nutrients in the body, compromising health and leading to increased morbidity and mortality (4). Malnutrition is frequently associated with head–neck malignant tumors and is a challenging condition that often requires invasive nutrition (5, 6). Tumors are the chronic condition with the highest incidence of malnutrition, affecting quality of life, healthcare costs, and survival, often preventing the possibility of drug or surgical treatment (7). Squamous cell carcinomas of the oral cavity (OSCC) rank as the second most common type of malignancy associated with malnutrition, following malignancies of the gastrointestinal tract (8, 9). This is due not only to the stage of the tumor but also to dysphagic symptoms that occur even before diagnosis, radiation therapy that may result in xerostomia, and subsequent resection surgeries that lead to functional alterations in mastication and swallowing (6, 10). Yeh and coll longitudinally investigated 50 patients with locally advanced head and neck squamous cell carcinoma demonstrating that at the same tumor stage, patients with malnutrition had a higher 3-year mortality rate than that of patients without malnutrition (52.9% vs. 15.2%) (11). Therefore, early comprehensive nutritional assessments and rigorous monitoring programs are recommended for specific oncological patients including those with head and neck tumors (12–14). Patients with OSCC may require invasive nutritional support such as nutrition via percutaneous endoscopic gastrostomy (PEG) or nasogastric tube (NG tube) even post-hospitalization, posing a considerable challenge for management in outpatient or community settings (15). Globally, malnutrition related to oncological diagnosis remains largely unrecognized, underestimated, and inadequately addressed in clinical practice (16); hence, the early diagnosis of malnutrition is a current challenge (17). For this purpose, suitable methods for measuring the amount of lean body mass are necessary. The bioimpedance analysis (BIA) is a frequently used method due to low cost, safety, high feasibility by non-specialized operators, and repeatability (18). However, BIA accuracy may be often compromised in hospitalized patients due to altered fluid distribution or fluid infusions (19). Imaging techniques, such as magnetic resonance (MRI) or computed tomography (CT) imaging, are powerful tools for investigating muscle and adipose tissue; however, these methods are still confined to research settings due to their costs and complexity of use (20).

Therefore, we retrospectively investigated if ON and AN, assigned by a dedicated nutritional team or not, influence survival in a cohort of patients with OSCC. As a secondary objective, in a subgroup of patients, we preliminary evaluated the MRI volumetric reconstruction of posterior paraspinal muscles at the level of the third cervical vertebra as a possible specific measure of the fat-free mass (MRI-C3).

## Materials and methods

### Participants

Patients who underwent surgery for OSCC at the Unit of Plastic Surgery of the University Hospital Policlinico “P. Giaccone” in Palermo (Italy) between January 2020 and May 2023, were retrospectively identified and included in this study. Briefly, starting in January 2020, a dedicated team of doctors and dieticians of the Unit of Clinical Nutrition, Obesity, and Metabolic Diseases offered specific nutritional support during hospitalization. Patients were classified according to the most recent definition of TNM stage (21). Upon discharge, nutritional assistance was taken care of by the local health services. Data collected were retrospectively compared with data retrieved from clinical records of a control group that consisted of patients surgically treated for OSCC analog oncological conditions between January and December 2019 in the same unit of surgery in the absence of a dedicated nutritional team. Moreover, MRI exams of those patients who were evaluated before and 3–12 months after surgery at the radiology unit of the same hospital were collected. Detailed medical history, anthropometric measurements, and laboratory data were recorded. After surgery, radiation or chemoradiation therapy was added as adjuvant treatment for high-risk patients. Systemic therapy could be used in cases of locoregional recurrence or distant metastases. Information concerning survival was obtained from medical records or by phone contact with patients or their care-givers.

All patients provided written consent for the use of their data for scientific purposes. The local Ethical Committee Palermo 1 approved the study protocol (reference # 13/2024 of the 22th May 2024). This study was conducted in accordance with the Declaration of Helsinki.

### Measurements

Anthropometric data such as height, body weight, body mass index (BMI, body weight (kg)/height^2^ (m^2^)), and mid-arm and calf circumferences (cm) were obtained. The Mini Nutritional Assessment (MNA) score for the screening of malnutrition was recorded. An MNA total score of <17 was indicative of malnutrition and a score of 17–23 was indicative of risk of malnutrition (22).

Body composition (fat mass, FM; fat-free mass, FFM) was determined when permitted by clinical conditions using bioelectrical impedance analysis with an 800 μA, 50 kHz tetrapolar impedance plethysmograph (BIA; BIA-101 Anniversary, Akern; Florence, Italy) and according to the manufacturer’s equations (23). Briefly, patients were evaluated in the morning after an overnight fast, in the supine position, and body resistance (R, Ohm), reactance (Xc, Ohm), and phase angle [PA degrees = arctan (Xc/R) × (180/*π*)] were measured. In some cases, BIA could not be performed due to compromised general clinical conditions, concomitant venous treatments, or difficulties in applying electrodes due to the presence of vein catheters. Images and reports of MRI were retrospectively analyzed. Briefly, T1-weighted sequences with and without fat saturation and intravenous gadolinium, T2-weighted, and STIR sequences were acquired in the axial and coronal planes, with a maximum slice thickness of 4 mm, using 1.5 T and 3 T MRI scanners (Ingenia 3.0 T, Philips Healthcare, Best, The Netherlands; Achieva 1.5 T, Philips Healthcare, Best, The Netherlands). Volumetric evaluation of the paravertebral muscles was performed by segmenting five slices at the C3 vertebral level on axial T2-weighted images using the 3D Slicer software (24). Manual segmentations were conducted by a radiologist with over 10 years of experience in MRI evaluation of the head and neck region. Muscle strength was measured as hand-grip strength using a hydraulic hand dynamometer (JAMAR SH5001; Saehan; Republic of Korea). Patients performed the test while sitting (if possible) and were requested to perform a maximal isometric contraction. Three tests were repeated at intervals of 15–20 s with each hand, and the average value (kg) of the three tests was used for the analysis (25). Each patient underwent routine blood tests during hospitalization using standard methods. The following serum parameters at first measurement were recorded for the purposes of the study: glucose, creatinine, albumin, total proteins, total cholesterol, white blood cells, hemoglobin, lymphocytes, iron, ferritin, folic acid, vitamin B12, vitamin D, sodium, potassium, magnesium, and phosphorus.

### Dietary assessment and support

Daily energy requirements of hospitalized patients following surgery were estimated as 30 kcal/kg body weight/day monitoring body weight and adjusting intake as required, and protein intake was 1.0–1.2 g/kg body weight/day (16, 26). On the basis of nutritional assessment, individualized nutritional support was prescribed, including dietary plans, oral supplements, enteral or parenteral nutrition, or combination strategy. The choice of the type of support was tailored to a single patient’s characteristics, needs, overall clinical features, and self-feed ability following current guidelines (14). In particular, in 2020–2023, industrially produced special medical food formulations were used rather than natural foods as it was in use in 2019; also, adequate protein supplements were assigned in order to reach the recommended protein intake. Furthermore, in 2020–23 the nutritional conditions of the patients were evaluated in most cases in the weeks preceding surgery, trying to improve nutritional status or prevent malnutrition with specific dietary suggestions and nutrient supplements. Upon discharge AN consisted exclusively in enteral nutrition.

### Statistical analysis

Assuming, for a three-year follow-up, a mortality (primary outcome of the study) of 30% in the group (probability of exposure 0.3) with dedicated nutritional intervention and 70% in the control group (probability of exposure 0.7), with an alpha error = 0.05 and a power = 80%, based on available enrollment periods of 1 year versus 3 years, a case:control enrollment ratio of 0,33, it was estimated a number of at least 10 patients in the control group and 40 in the case group^1^. Patients were categorized based on TNM staging (stage A: I, II, and III; stage B: IVa, IVb, and IVc) and nutritional practices at discharge (oral nutrition, ON; artificial nutrition AN). Data are presented as means ± SD for continuous variables and as percentages for categorical variables. All variables with skewed distribution were log-transformed, changed to normal distribution and analyzed using parametric tests and then back transformed. Student’s *t*-test or Pearson’s chi-square test was applied to compare between-group differences. Kaplan–Meier survival curves were constructed and outcome differences were evaluated using the log-rank test. Multivariate Cox regression stepwise analysis was applied to determine the independent factors associated with mortality. Hazard ratios with 95% confidence intervals were presented. Simple linear correlation analysis with calculation of *r* coefficients investigated the association between variables. Cases with incomplete body composition data were excluded from calculations. A *p*-value of <0.05 was considered statistically significant. All statistical analyses were performed using Systat (Windows version 13.0; San Jose, CA, USA).

## Results

**Figure 1:**
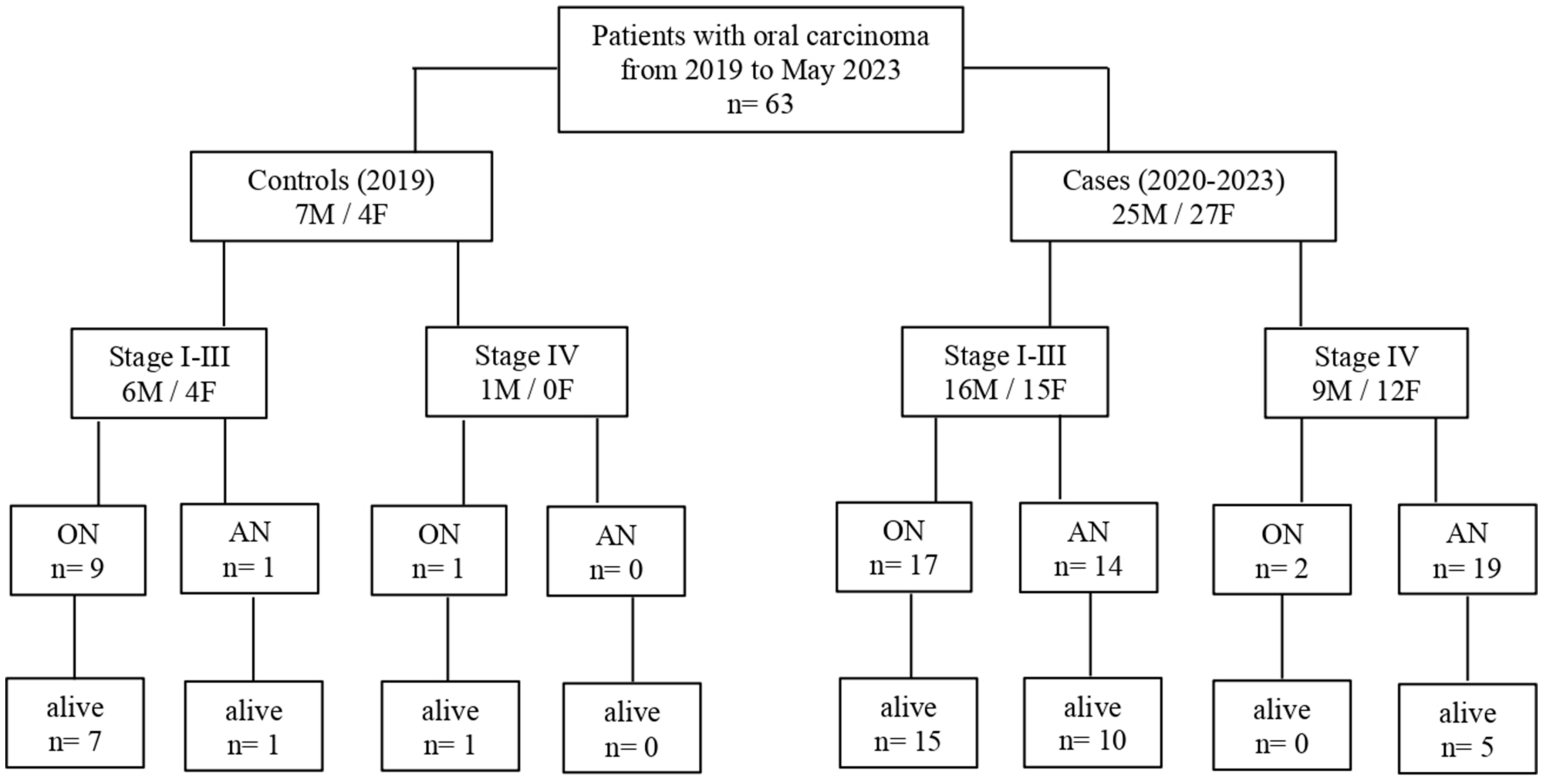
Flowchart of participant retrospective recruitment according to different nutritional support assigned by a dedicated team (2020–2023) or not (2019) (ON, oral nutrition; AN, artificial nutrition; and stage: TNM stage).

**Figure 2:**
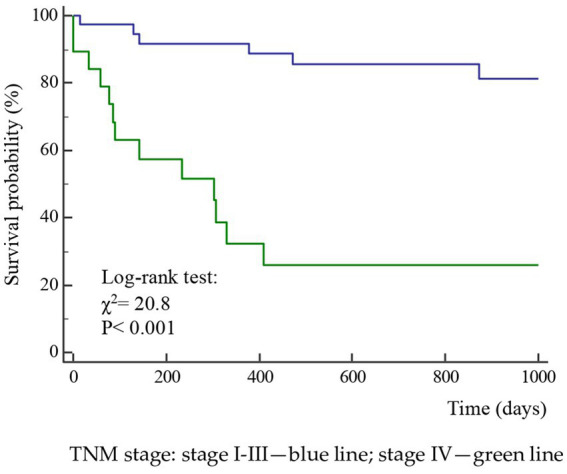
Kaplan–Meier survival probability curves according to the TNM stage of the disease in 63 patients after surgery for squamous cell carcinomas of the oral cavity.

**Figure 3:**
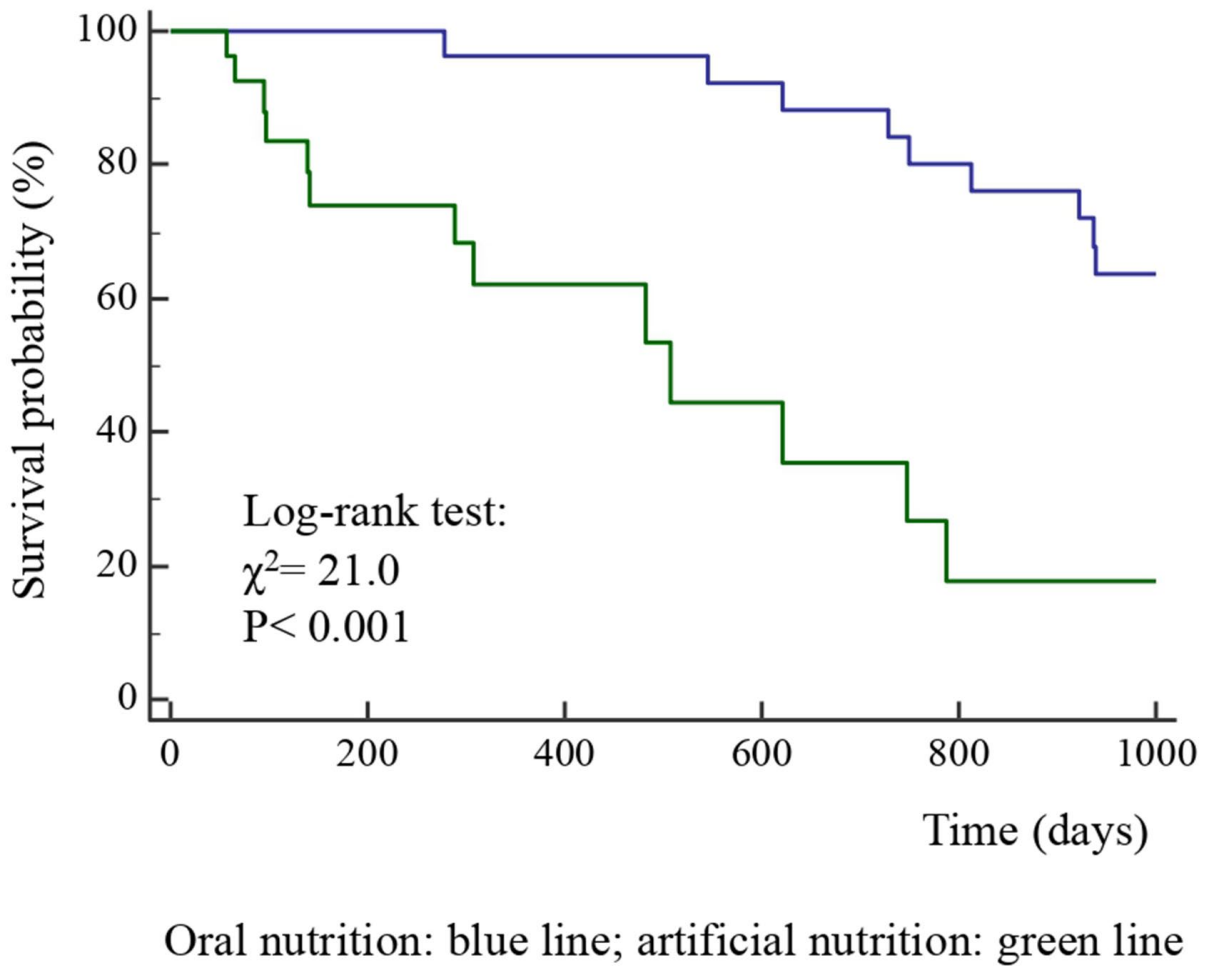
Kaplan–Meier survival probability curves according to nutrition type (ON, oral nutrition; AN, artificial nutrition) in 63 patients after surgery for squamous cell carcinomas of the oral cavity.

**Figure 4:**
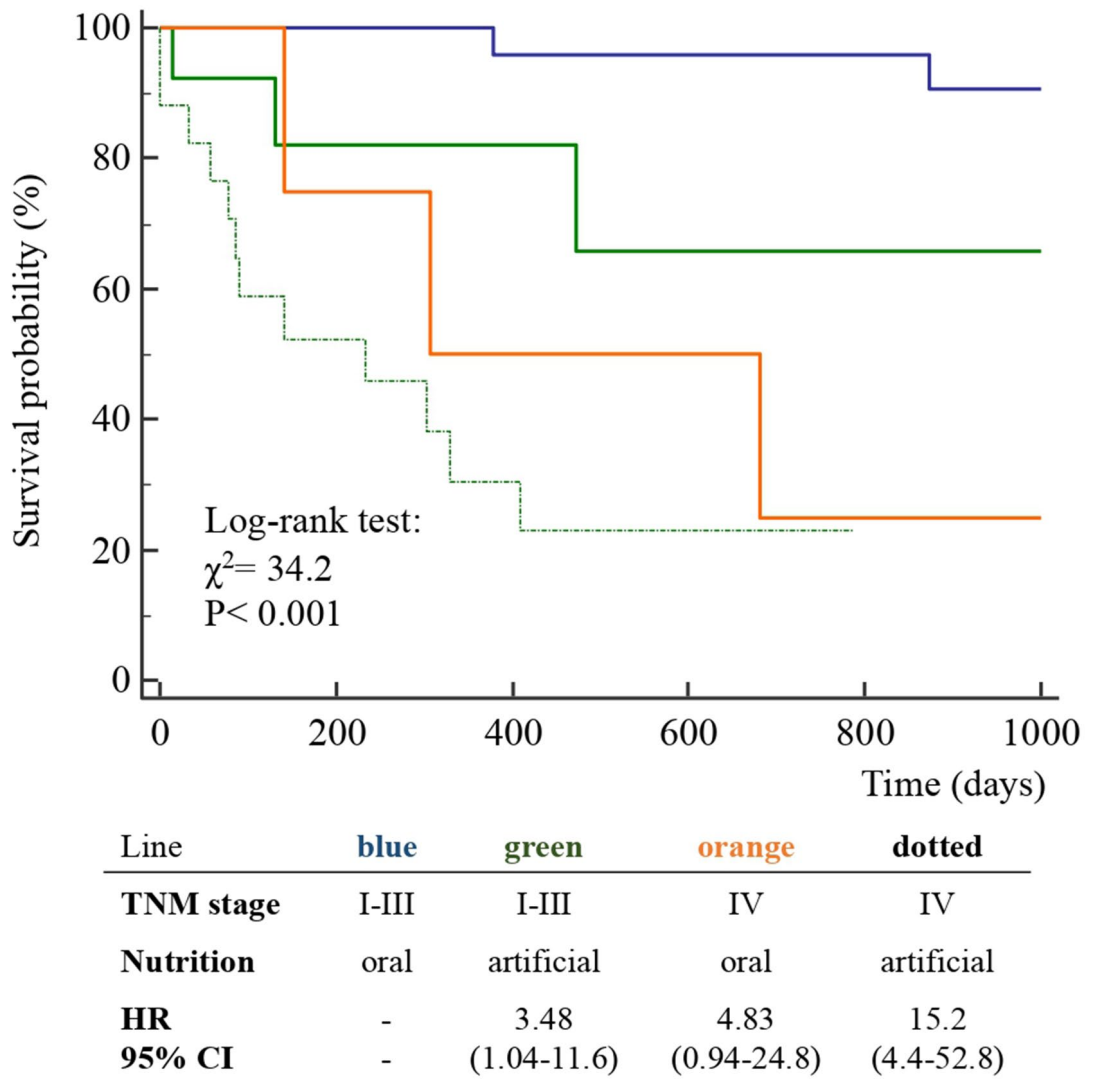
Kaplan–Meier survival probability curves of the participants classified according to TNM stage and nutrition type (ON, oral nutrition; AN, artificial nutrition) in 63 patients after surgery for squamous cell carcinomas of the oral cavity.

**Figure 5:**
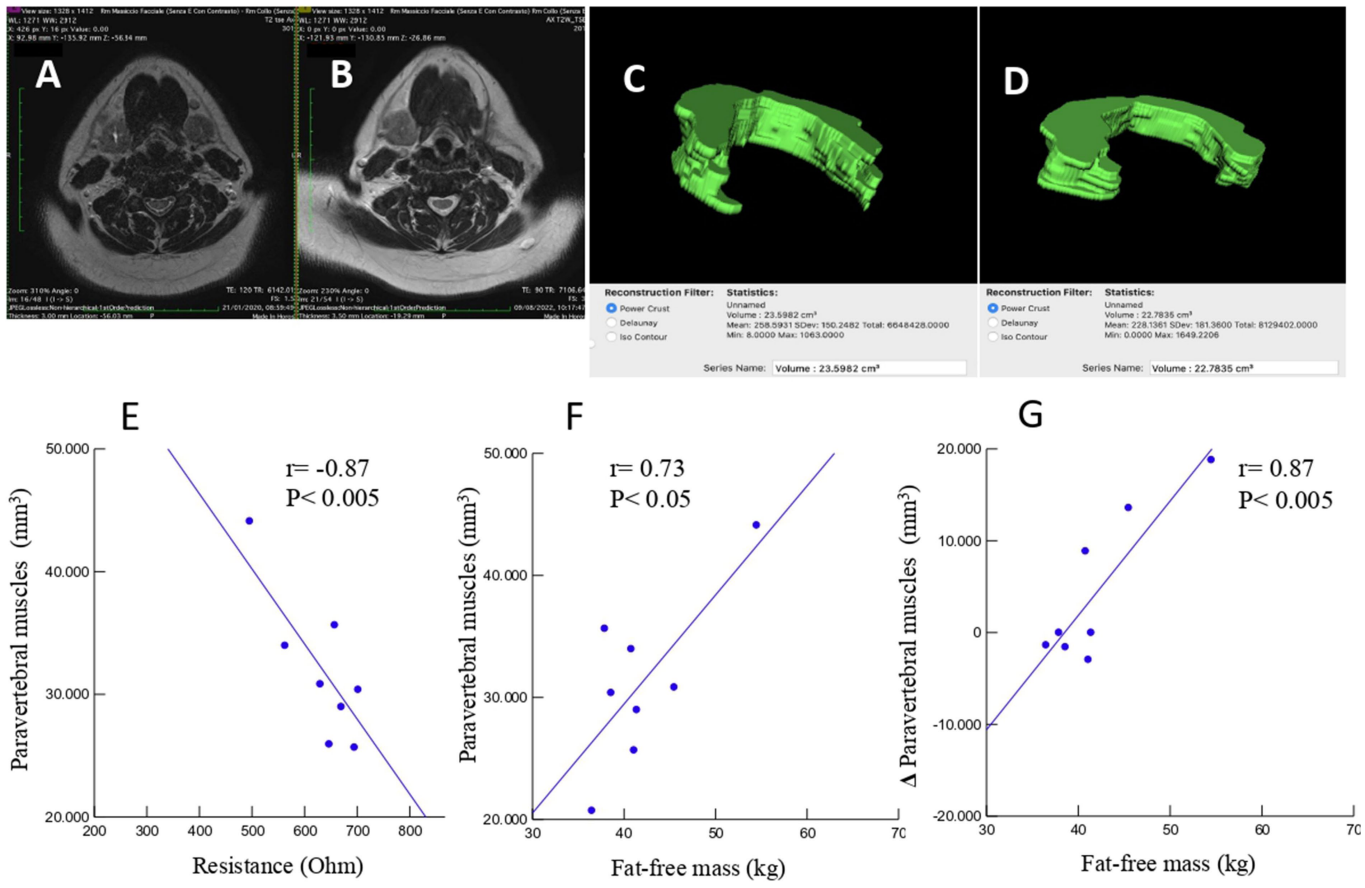
An example case of a 65-year-old male patient undergoing head–neck MRI before **(A)** and 8 months after **(B)** surgical intervention for right hemi-glossectomy due to OSCC of the tongue. Volumetric reconstructions and measurement of the volume of the posterior neck muscles at C3 level, in 5 slices, before **(C)** and after **(D)** intervention demonstrating a volumetric reduction (23.5982 cm^3^ vs. 22.7835 cm^3^). Linear regression analysis of cases with volumetric reconstruction of paravertebral muscle volume and bioelectrical resistance **(E)**, fat-free mass **(F)**, and change in paravertebral muscle volume and fat-free mass **(G)**.

A total of 52 cases and 11 controls were selected. The participant selection flowchart is shown in Figure 1. The two groups were not comparable; in fact, the control group included a higher prevalence (85.7% vs. 59.6%) of less severe cases (TNM I-III) with higher survival rates at follow-up than the case group (80.5% vs. 27.3%). Given the limited number of cases, all patients were included in a unique group. Physical, clinical, and nutritional characteristics of patients are reported in Tables 1, 2. Among the variables considered, age, body weight, FFM, FFM index and serum creatinine had a non-normal distribution. The mortality rate was significantly higher in the group with severe TNM stage (Table 1) and in patients on artificial nutrition (Table 2). The Kaplan–Meier survival analysis demonstrated that TNM I-III (Figure 2) and ON (Figure 3) had a higher survival probability that progressively reduced through the TNM stage and the modality of nutrition in different combinations from TNM I-III + ON to TNM IV + AN (Figure 4). The survival probability was not significantly different according to the presence of a MNA score indicative of malnutrition or risk of malnutrition (*χ*^2^ = 0.03; *p* = non-significant -ns-), or of BMI < 18 kg/m^2^ (*χ*^2^ = 0.79; *p* = ns). A significantly lower survival probability was found for serum albumin concentrations <3.5 g/dL (*χ*^2^ = 12.4; *p* < 0.001) and serum creatinine concentrations <0.7 mg/dL (*χ*^2^ = 6.51; *p* < 0.01). Cox proportional hazard regression (stepwise) analysis including TNM stage A vs. B, nutrition ON vs. AN, serum creatinine <0.7 mg/dL, albumin <3.5 g/dL, BMI <18.0 kg/m^2^, sex and age < 70 years as categorical variables demonstrated an independent effect (*χ*^2^ = 20.8; *p* < 0.001) on the outcome of death according to TNM stage (IV vs. I-III; *p* < 0.05; HR, 3.67; 95% CI, 1.05–12.9) and nutrition modality (AN vs. ON; *p* < 0.05; HR, 4.98; 95% CI, 1.07–23.2). The change in MRI_C3 (Δ MRI_C3) 3–12 months after surgery was obtained in a subsample of 16 patients; the results showed that MRI_C3 increased significantly in ON-treated patients and decreased in patients receiving AN treatment (Table 2); moreover, MRI_C3 was not significantly different between patients with TNM I-III and TNM IV (1803 ± 7,201 mm^3^ vs. −148 ± 8,863 mm^3^; *p* = 0.64). The MRI_C3 before surgery was significantly correlated with the FFM-kg (*r* = 0.73; *p* < 0.05), BIA resistance (*r* = −0.87; *p* < 0.005), and reactance (*r* = −0.78; *p* < 0.05); the change in MRI_C3 was correlated with the FFM-kg (*r* = 0.87; *p* < 0.005) (Figure 5).

**Table 1:** Physical, clinical, and nutritional characteristics of the cohort of patients with oral squamous cell carcinoma according to TNM stage.

	Oral cancer stage		
	I-III *n* = 41	IV *n* = 22	*p*^a^
Males (%)	53.7	45.5	0.54
Age (years)	65 ± 17	64 ± 11	0.85
Actual survival from surgery (days)	764 ± 465	317 ± 360	< 0.001
All-cause death (%)	19.5	72.7	< 0.001
Nutrition (%):			
Oral	63.4	13.6	
Artificial	36.6	86.4	< 0.001
Body weight (kg)	68.0 ± 17.5	60.3 ± 13.2	0.07
Body mass index (kg/m^2^)	25.3 ± 5.4	22.2 ± 5.8	0.06
Bioimpedance analysis:	*n* = 11	*n* = 12	
Resistance (Ohm)	641 ± 114	544 ± 142	0.08
Reactance (Ohm)	65 ± 21	44 ± 16	< 0.01
Phase angle (°)	5.7 ± 1.1	4.5 ± 0.9	< 0.01
Fat mass (%)	26.6 ± 10.3	17.5 ± 10.2	0.06
Fat-free mass (kg)	44.2 ± 8.7	45.6 ± 8.4	0.70
Fat-free mass index (kg/m^2^)	16.8 ± 2.3	16.8 ± 3.2	0.98
Hand-grip test (kg):	*n* = 12	*n* = 14	
Right	21.3 ± 9.7	23.3 ± 11.4	0.64
Left	20.2 ± 10.2	21.1 ± 10.1	0.82
Mini nutritional assessment (%):			
Risk of malnutrition	47.1	38.9	
Malnutrition	52.9	61.1	0.54
Serum concentration of:			
Proteins (g/dl)	6.1 ± 1.0	5.4 ± 0.8	< 0.005
Albumin (g/dl)	3.8 ± 0.6	3.3 ± 0.7	< 0.01
Hemoglobin (g/dl)	11.0 ± 2.3	10.2 ± 2.2	0.25
Creatinine (mg/dl)	0.74 ± 0.24	0.69 ± 0.42	0.61

Mean ± SD; ^a^unpaired Student’s *t*-test or *χ*^2^ when appropriate.

**Table 2:** Physical, clinical, and nutritional characteristics of the cohort of patients with oral squamous cell carcinoma according to modality of nutrition.

	Nutrition		
	Oral *n* = 29	Artificial *n* = 34	*p*^a^
Males (%)	65.5	38.2	< 0.05
Age (years)	64 ± 18	66 ± 12	0.55
Actual survival from surgery (days)	994 ± 353	289 ± 299	< 0.001
All-cause death (%)	20.7	52.9	< 0.01
TNM stage I-III (%)	89.7	44.4	< 0.001
Body mass index (kg/m^2^)	26.4 ± 5.7	22.5 ± 5.1	< 0.01
Bioimpedance analysis:	*n* = 6	*n* = 17	
Phase angle (°)	5.9 ± 0.9	4.8 ± 1.1	< 0.05
Fat-free mass index (kg/m^2^)	17.4 ± 2.5	16.6 ± 2.8	0.53
MRI_C3 (mm^3^):	*n* = 9	*n* = 7	
Before surgery	35,586 ± 5,063	28,728 ± 6,151	< 0.05
After (3–12 months) surgery	44,822 ± 10,685	26,088 ± 5,123	< 0.001
Δ MRI_C3	9,236 ± 7,313	−2,640 ± 4,184	< 0.001
Serum creatinine (mg/dl)	0.79 ± 0.23	0.67 ± 0.35	0.14

Mean ± SD; ^a^unpaired Student’s *t*-test or *χ*^2^ when appropriate.

MRI_C3, volumetric magnetic resonance imaging of posterior paravertebral muscles at the C3 level.

## Discussion

This study agrees with previous investigations demonstrating that OSCC patients often develop malnutrition even before surgical treatment, a condition that may reduce the possibilities of effective therapy (27). In fact, we observed a high prevalence of malnutrition or risk of malnutrition in the TNM B group and, following surgery, invasive nutrition was prescribed in the majority of these patients (86.4%). However, in patients with a lower stage of the disease (TNM A group), we also observed a high prevalence of malnutrition, which suggests that patients with OSCC experience difficulties in consuming food independently from the stage of the disease. The diagnosis of malnutrition is challenging in patients with OSCC and we confirm (26) that many traditional indicators of malnutrition, such as BMI, fat mass, fat-free mass index, MNA, hemoglobin, creatinine, or hand-grip test, may not be adequate in this particular cancer (Table 1). On the other hand, measurements such as bioelectrical reactance and phase angle or serum proteins and albumins were significantly altered in the TNM B group with respect to the TNM A group. On account of the difficulties in obtaining the traditional measures indicative of malnutrition in all patients (19), new diagnostic tools need to be defined for use in people with head–neck tumors that are also in support of the BIA method, which may present some limitations in admitted patients (19). In fact, patients undergoing surgery for OSCC often require complex reconstructive procedures, including skin and muscle grafts taken from the limbs, with consequent difficulty in positioning electrodes. The BIA measurements may also be altered due to the presence of localized edema of the neck and upper limbs consequent to lymphadenectomy (23). Currently, computed tomography (CT) and DEXA techniques are reference methods but rarely accessible. In particular, the CT is the gold standard method for assessing muscle mass through the muscle mass index calculated from the L3 vertebra (28); however, its application is also limited by the exposure to large amounts of X-rays since in many patients the CT of the abdomen is not requested for staging the disease or for follow-up purposes. Ultrasound has the intrinsic limitation of being an operator-dependent technique with low reproducibility. Recently, Fernandez-Jimenez et al. (29) found that different ultrasound measurements of the quadriceps rectus femoris muscles were associated with malnutrition in patients with head–neck cancer, but the predictive value of the probability of survival was rather low. Interestingly, it was recently proposed to evaluate the cross-sectional area of the neck muscles at C3 as a marker of sarcopenia (30–32). In the present study, we tried to develop an alternative technique using MRI to measure the volume of paravertebral muscles at that level. This approach may present some considerable advantages. In fact, an MRI of the neck is always available in these patients, even at follow-up. Furthermore, the paravertebral muscles are used in any case and are not expected to be altered by potential confounding factors such as atrophy secondary to muscular inactivity, as it may occur for muscles of the lumbar region or lower limbs of patients forced into inactivity in bed or in an armchair. In previous studies (31, 32), all areas of the muscles of the neck were segmented as a single slice at the level of the C3 vertebra. However, patients with OSCC typically undergo radical or modified radical neck dissection, which includes sternocleidomastoid muscle removal in the most advanced stages. Therefore, we excluded sternocleidomastoid muscle segmentation generating a widely applicable MRI system for investigating differences during follow-up. To the best of our knowledge, this is the first study to focus exclusively on the paraspinal muscles, which are usually preserved by surgery and are less affected by reduced physical activity or bedridden syndrome. Indeed, the ergonomics of neck muscles is to be considered. In fact, in the upright position, the head is balanced and moves on the neck, with the gravity vector largely parallel to the neck. In the clinostat position, as during bed rest, the gravitational forces are predominantly perpendicular to the neck, which means that greater muscle strength is required to lift and move the head (33), implying a surplus of exercise for this muscle compartment that may contribute to preserving it more than other muscles. Moreover, we segmented the MRI images of the paraspinal muscles of the neck in five slices at the C3 vertebra level and calculated the muscle volume. Therefore, moving from bi-dimensional to three-dimensional segmentation is expected to improve accuracy (33, 34). Interestingly, in the small subsample of patients who also had BIA values available (Figure 5), highly significant correlations were observed between the volume of paravertebral muscles at C3 and the values of resistance and fat-free mass. We obtained MRI_C3 measurements in only 16 patients and observed that, before surgery, those who underwent AN treatment had significantly lower values than patients receiving ON treatment, thus suggesting a higher prevalence of sarcopenia (Table 2).

The diagnosis of malnutrition is of great importance in patients with OSCC as it may affect the survival and the adoption of the most appropriate nutritional strategies. As expected, the TNM stage significantly influenced the probability of survival (Figure 2); however, surprisingly, the AN was independently associated with a lower probability of survival than the ON (Table 2, Figures 3, 4). This result may indicate that AN is associated with a more advanced stage of the disease but also that patients receiving AN have a higher prevalence of malnutrition as the BMI or phase angle value would suggest (35) (Table 2). We cannot exclude that the nutritional follow-up of patients receiving AN treatment would have requested special attention following discharge. In fact, the MRI_C3 3–12 months after surgery increased in patients receiving ON treatment but reduced in those receiving AN, suggesting that malnutrition worsened in the latter group after surgery. Managing oncologic patients is challenging during hospitalization and likely even more so after discharge (14). Nutrition home management in oncological patients, especially for invasive nutrition, is strongly influenced by territorial healthcare organizations and the socioeconomic status of patients. For specific oncological patients, such as those with head and neck cancers, the importance of structuring comprehensive nutritional assessments and rigorous monitoring programs is emphasized (14). Our results contribute to highlighting these serious nutritional problems and the need for dedicated professionals to care for them during hospitalization and after discharge (16).

In the present study, males and females were equally affected by OSCC; this result is in agreement with the current progressive reversal of epidemiological trends, probably due to the increasing habit of smoking in women (36). However, two-thirds of patients receiving the artificial nutrition treatment were female, suggesting a higher prevalence of malnutrition and more severe cases in women; this gender difference remains to be investigated.

This study has intrinsic limitations. First, the aim of this study was to organize a longitudinal retrospective case–control study; however, we could not obtain an adequate control group. This aspect is mainly due to the fact that the unit of surgery where patients were enrolled treated less serious cases in the past and subsequently opened up to more complex cases; therefore, we could not obtain a real historical control group. However, the probability of organizing a real case–control intervention study is low since prospective, longitudinal studies are basically unethical, and retrospective studies have low probabilities of recruiting a real control group as evidenced in this study. Another limitation was the absence of data at follow-up apart from those concerning survival. This fact suggests the need for a more stringent collaboration between hospital and territorial systems of assistance, which is a matter that is not easy to implement given the particular gravity of the disease that in many instances requests long-term institutionalizations even far from the place of care. Also, the small sample size for the MRI-C3 subgroup may impact the statistical power of related conclusions. Finally, this was a monocentric study with a limited number of cases and fragmented data; in future studies, an adequate network between different centers is required in order to collect and share data that may contribute to a better understanding of the specific role of nutritional factors and the implementation of strategies of treatment.

## Conclusion

Despite significant methodological limitations, this study demonstrates that malnutrition, or risk of malnutrition, is almost invariably associated with oral OSCC, often precedes surgical treatment, requires AN in a high percentage of cases, and that the latter is independently and unfavorably associated with survival. The MRI measurement of the volume of paraspinal muscles at the C3 level is a promising technique for diagnosing and monitoring sarcopenia that needs to be confirmed by further studies including larger groups of patients. There is a need for teams dedicated to the nutritional management of patients with OSCC, as this condition may influence patient prognosis and quality of life.

## Data Availability

The raw data supporting the conclusions of this article will be made available by the authors, without undue reservation.

## Footnotes

^1^ https://riskcalc.org/samplesize/
